# Photoconversion Mechanism at the *pn*-Homojunction Interface in Single Organic Semiconductor

**DOI:** 10.3390/ma13071727

**Published:** 2020-04-07

**Authors:** Ji-Hyun Lee, Armand Perrot, Masahiro Hiramoto, Seiichiro Izawa

**Affiliations:** 1 Institute for Molecular Science, 5-1 Higashiyama, Myodaiji, Okazaki, Aichi 444-8787, Japan; leejihyun@ims.ac.jp (J.-H.L.); armand.perrot@etu.chimieparistech.psl.eu (A.P.); hiramoto@ims.ac.jp (M.H.); 2 SOKENDAI, The Graduate University for Advanced Studies, 5-1 Higashiyama, Myodaiji, Okazaki, Aichi 444-8787, Japan; 3 École Nationale Supérieure de Chimie de Paris, 11 Rue Pierre et Marie Curie, 75005 Paris, France

**Keywords:** organic solar cell, *pn* junction, interface, doping, single organic semiconductor, photoconversion

## Abstract

Clarifying critical differences in free charge generation and recombination processes between inorganic and organic semiconductors is important for developing efficient organic photoconversion devices such as solar cells (SCs) and photodetector. In this study, we analyzed the dependence of doping concentration on the photoconversion process at the organic *pn*-homojunction interface in a single organic semiconductor using the temperature dependence of *J–V* characteristics and energy structure measurements. Even though the organic *pn*-homojunction SC devices were fabricated using a single host material and the doping technique resembling an inorganic *pn*-homojunction, the charge generation and recombination mechanisms are similar to that of conventional donor/acceptor (D/A) type organic SCs; that is, the charge separation happens from localized exciton and charge transfer (CT) state being separated by the energy offset between adjacent molecules, and the recombination happens from localized charge carrier at two adjacent molecules. The determining factor for photoconversion processes is the localized nature of charges in organic semiconductors. The results demonstrated that controlling the delocalization of the charges is important to realize efficient organic photoconversion devices.

## 1. Introduction

Free charge generation processes by light absorption in inorganic and organic semiconductors are completely different. Inorganic semiconductors have a large dielectric constant; thus, free charges are directly formed after the light absorption in a single semiconductor material [1]. In contrast, a strongly bounded Frenkel type exciton forms after light absorption in organic semiconductors, because they generally have a smaller dielectric constant when compared with that of inorganic semiconductors [2]. The exciton can be separated using the energy offset between the two organic semiconductor materials called donor and acceptor in organic solar cells (OSCs) [2,3]. The question is whether free charge formation is possible in single organic semiconductor material by light absorption, similar to that in inorganic semiconductors.

Recently, we have reported that *pn*-homojunction interfaces in a single organic semiconductor films formed by doping can achieve efficient charge separation [4]. The organic *pn*-homojunction solar cell SC device showed a high internal quantum efficiency of 30%. Further investigation for clarifying the mechanism of the photoconversion at the *pn* homojunction interface can help to answer the basic question of how free charges form beyond the strong coulomb binding and recombine in a single organic semiconductor film, and what is the critical difference in these processes between organic and inorganic semiconductors.

In this study, we analyzed the doping concentration dependence on the photoconversion process in organic *pn*-homojunction SC devices mainly by a temperature dependence measurement. The measurement gives information about thermal activation for the charge generation and recombination pathways [5,6]. Upon combining an energy structure measurement near the *pn*-homojunction, we revealed the detailed mechanism of photoconversion at the single organic semiconductor interface.

## 2. Results and Discussion

The *pn*-homojunction SC devices were fabricated in the same manner as previously reported [4]. We used diindenoperylene (DIP), which is an ambipolar organic semiconductor molecule, as the host, and MoO_3_ and Cs_2_CO_3_ as *p*- and *n*-dopants, respectively. The device was fabricated by thermal evaporation under high vacuum, and the structure was indium tin oxide (ITO)/MoO_3_: 10 nm/MoO_3_-doped DIP: 50 nm/Cs_2_CO_3_-doped DIP: 50 nm/bathocuproine (BCP): 10 nm/Al: 60 nm (Figure S1). Dopants were introduced into the semiconductor layer via co-deposition techniques and the concentration of the dopants relative to the semiconductor volume was controlled by varying the ratio between the deposition rates of the two species [4]. Figure S2 shows the typical *J–V* characteristics of *pn*-homojunction devices and the performances are summarized in Table S1. As previously reported, *J*_SC_ drastically increased and *V*_OC_ decreased with an increase in doping concentration. The degrees of *J*_SC_ increase were eight times from undoped to 5% doping in the DIP devices. In contrast, *V*_OC_ decreased from 1.13 V to 0.83 V. The fill factor (FF) values increased marginally in the device with high doping concentration. The increase of external quantum efficiency (EQE) spectra without the change in spectral shape (Figure S3) indicated that charge separation from the absorption of the ground state of DIP was accelerated by doping.

**Figure 1:**
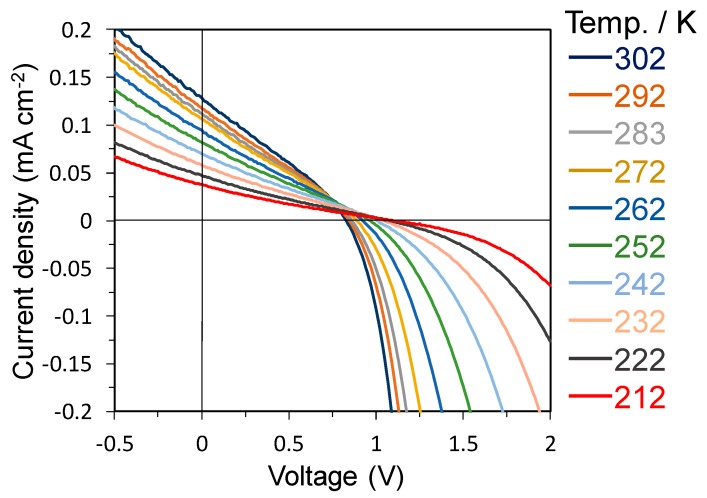
*J–V* curves for the 5% doped *pn-*homojunction device at various temperatures under AM 1.5 irradiation (100 mW cm^−2^).

To investigate the charge generation and recombination processes, we measured the *J–V* characteristics of the devices at temperatures from 30 to −60 °C, as shown in Figure 1.

**Figure 2:**
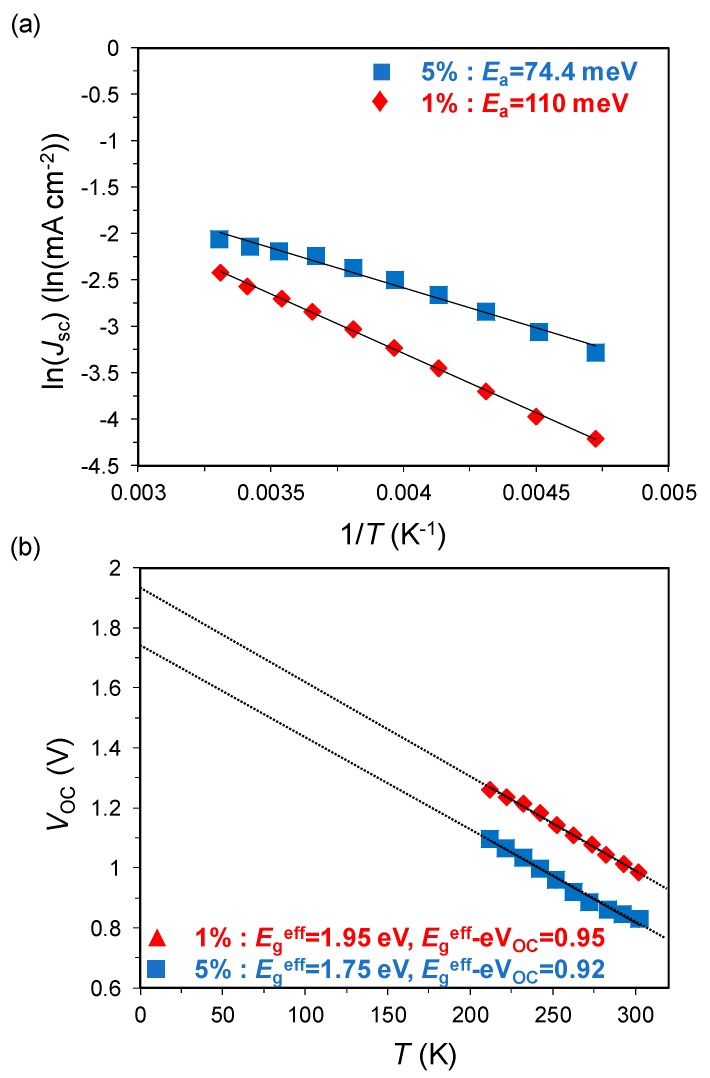
Temperature dependence of (**a**) *J*_SC_ and (**b**) *V*_OC_ for the 1% doped (red) and 5% doped (blue) *pn-*homojunction devices.

Typically, *J*_SC_ decreases while *V*_OC_ increases with a decrease in temperature in the OSCs [6]. The *pn*-homojunction devices followed the same tendency. Firstly, the temperature dependence of *J*_SC_ is expressed by the following Arrhenius Equation (1):

*J*_SC_ = *J*_0_(*P*_light_)exp(−*E*_a_/*kT*), (1)

where *J*_0_(*P*_light_) is the pre-exponential factor, *E*_a_ is the activation energy, *k* is Boltzmann constant, and *T* is temperature [7]. The origin of *E*_a_ was attributed to the activation process during charge separation [5,8]. The calculated *E*_a_ of the devices with 5% and 1% doped DIP from the Arrhenius plots in Figure 2a are 74.4 and 110 meV, respectively.

The 5% doped device showed a lesser *E*_a_ than 1% doped, indicating that the charge separation was accelerated in the device with high doping concentration.

Next, the temperature dependence of *V*_OC_ is expressed by the following equation:

*qV_OC_* = *E_g_^eff^* + *nkTln*(*J_ph_/J_00_*), (2)

where *q* is elementary charge, *E*_g_^eff^ is effective bandgap energy at the interface, *n* is ideality factor, *J*_ph_ is the photogenerated current density, and *J*_00_ is the pre-exponential factor of the reverse saturation current density [9]. Equation (2) represents the *V*_OC_, which is determined by mainly by two parts, that is, the former energetic term and latter recombination loss term, which are dependent on the temperature [6]. The *V*_OC_ plots as a function of temperature are shown in Figure 2b. *E*_g_^eff^ of the devices with 5% and 1% doped DIP calculated from the intercept of the plots are 1.75 and 1.95 eV, respectively. On the other hand, the *V*_OC_ loss values induced by the recombination *E*_g_^eff^/*q* − *V*_OC_ of both devices showed similar values of 0.95 and 0.92 V, respectively. The results indicate that the decrease in *V*_OC_ owing to an increase in doping concentration is the result of the reduction in the effective energy gap at the interface, not because of the difference in the recombination processes.

**Figure 3:**
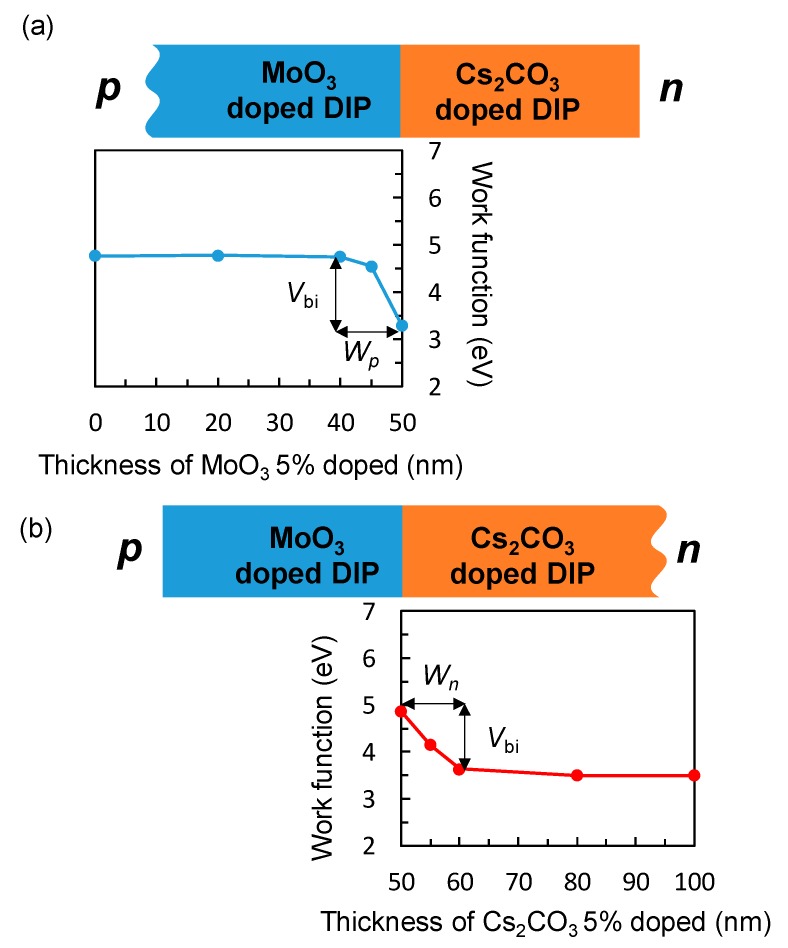
Work function (WF) of (**a**) 5% MoO_3_-doped and (**b**) 5% Cs_2_CO_3_-doped diindenoperylene (DIP) films on the Cs_2_CO_3_-doped film and MoO_3_-doped film, respectively, as a function of the thickness.

The energetic structure of the doped DIP films was estimated by energy-level mapping using the Kelvin probe (KP) method to measure the work function (WF) [10,11,12]. Figure 3 shows the schematic image of the KP measurement and the result of the 5% doped DIP film.

The results of the 1% doped DIP film are shown in Figure S4. The WF of the *p*-doped layer was measured on an Al/BCP/*n*-doped layer substrate and that of *n-*doped layer was measured on an ITO/MoO_3_/*p*-doped layer substrate. The steep change in the WF near the *pn*-interface was observed in all of the films. The ionization potentials (IP) of the undoped and doped DIP films were evaluated by photoelectron yield spectroscopy (PYS) in Figure S5. The IPs of the undoped and doped films did not change significantly, indicating that doping does not affect the IP of the films. Small changes in the absorption spectra (ABS) shown in Figure S3b also indicate that the bandgap and electron affinity (EA) of the film were not affected by doping. Therefore, the energetic structure of doped DIP films was determined only by the WF difference [10]. 

The energy diagrams of the active layer in the *pn*-homojunction devices were estimated based on the result of the KP measurement [13]. The drawback of the KP measurement is that it can only measure the WF at the surface of the films. We cannot observe the WF change of the underlying layer after the deposition of the over layer. To estimate the energetic structure of whole films, we calculated the electric potential distribution derived from Poisson’s equation [13]. The total depletion layer width (*W*) of *pn*-junction for uniformly doped semiconductor is given by the following equation:

(3) $$W=\sqrt{\frac{2\varepsilon_{r}\varepsilon_{0}V_{bi}\left(N_{p}^{-}+N_{n}^{+}\right)}{qN_{p}^{-}N_{n}^{+}}},$$

*qN*_n_^-^*x*_p_ = *qN*_n_^+^*x*_n_, (4)

where *ε*_r_, *ε_0_*, *V*_bi_, *N*_n_^−,^ *N*_p_^+^, *x*_p_, and *x*_n_ denote the relative dielectric constant of the semiconductor, the dielectric permittivity in a vacuum, the built-in-potential, ionized dopant concentration of the *p* and *n* layer, and the depletion layer width in the *p*- and *n-*type regions, respectively. The *N*_p_^+^ and *N*_n_^−^ values can be calculated by the measured *x*_p_, *x*_n_, and *V*_bi_ values in Figure 3 and Figure S4. The electric potential distribution *V*(x) at the *pn*-junction can be found by integrating Poisson’s equation as follows:

(5) $$V\left(x\right)=-\frac{qN_{n}^{+}}{2\varepsilon_{r}\varepsilon_{0}}{\left(x+x_{n}\right)}^{2}+V_{bi}\;\left(-x_{n}≦x≦0\right),$$

(6) $$V\left(x\right)=\frac{qN_{p}^{-}}{2\varepsilon_{r}\varepsilon_{0}}{\left(x_{p}-x\right)}^{2}\;\left(0≦x≦x_{p}\right),$$

**Figure 4:**
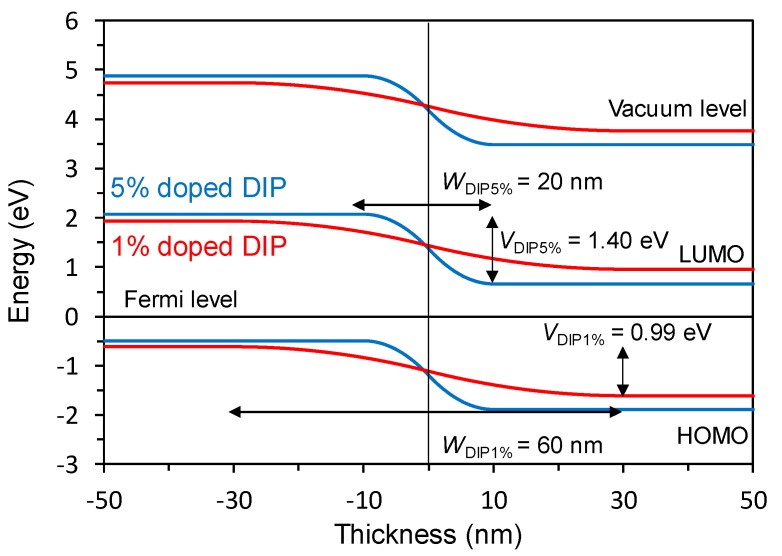
Vacuum level, HOMO, and LUMO energy levels relative to the Fermi level in the 1% doped DIP (red) and 5% doped DIP (blue) devices as a function of film thickness. These results are based on the KP measurement shown in Figure 3 and Figure S4 and the calculation by Equations (5) and (6).

Figure 4 shows the calculated energy diagram of the active layer in the *pn*-homojunction devices.

The *W* and *V*_bi_ values of the 5% and 1% doped DIP devices are 20 nm and 1.40 eV and 60 nm and 0.99 eV, respectively. This shows that the higher doping concentration decreased the *W* value and increased the *V*_bi_ value.

**Figure 5:**
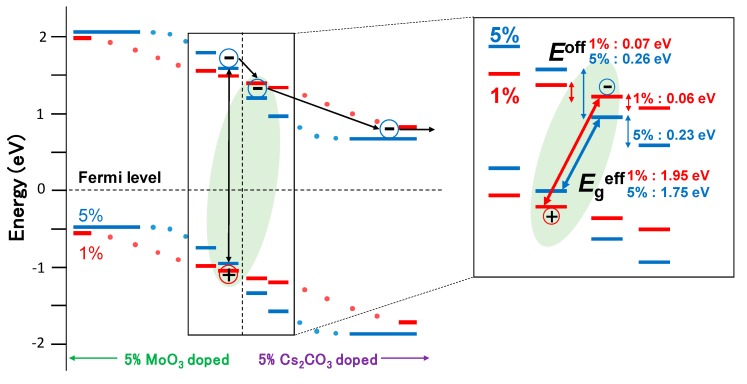
Schematic energy diagram and charge dissociation and recombination mechanisms in organic *pn-*homojunction SCs. The inset shows the energy gap between adjacent molecules at the *pn-*interface with 1% (red) and 5% (blue) doping.

The charge generation and recombination processes in the *pn*-homojunction devices are discussed based on the results of the temperature dependence of *J–V* characteristics and the energy structure measurements. Firstly, *J*_SC_ increased eight times owing to doping and *E*_a_ for charge separation decreased from 110 meV in the 1% doped *pn*-homojunction devices to 74.4 meV in the 5% doped *pn*-homojunction devices. In this study, we used the same host material, and only the interfacial energetics were modified by doping. The light intensity dependence of *J*_SC_ in the 1% and 5% doped devices in Figure S6 showed that the slopes of both the devices were close to unity, indicating that the bimolecular recombination is negligible under a short circuit condition [14]. Thus, the origin of the difference in the *E*_a_ induced by doping is attributed to the charge separation process and not to the exciton diffusion, nor the bimolecular recombination process. The charge separation processes are mainly separated into the two processes: exciton to CT state, which is defined as a bounded charge pair by the coulomb attraction at the interface, and the CT state to the charge separated state [2]. The energy structure measurement revealed that the energy offset between adjacent molecules at the *pn*-interface was 0.07 and 0.26 eV for 1% and 5% doped devices, respectively. The offset at one molecular layer apart from the *pn*-interface was 0.06 and 0.22 eV in the 1% and 5% doped devices, respectively (Figure 5). 

The conventional donor/acceptor (D/A)-type OSCs have an energy offset only at the D/A interface, and an offset larger than 0.3 eV not only accelerates the exciton dissociation, but also suppresses geminate recombination from the CT state [15]. By the analogy with the D/A-type OSCs, the energy offset between adjacent molecules at the *pn*-interface is crucial for charge separation in the *pn*-homojunction devices. The reason for the smaller *E*_a_ in the 5% doped device, compared with that in the 1% device, was that larger energy offset close to 0.3 eV in the 5% doped device not only accelerated exciton dissociation, but also suppressed geminate recombination from the CT state.

In contrast to *J*_SC_, *V*_OC_ decreased from 0.98 V in the 1% doped device to 0.83 V in the 5% doped device. Temperature dependence of *V*_OC_ revealed that the *V*_OC_ difference in the doping concentration was attributed to the *E*_g_^eff^ difference, not to the recombination loss difference. *E*_g_^eff^ reflects the energy of charge recombination center [16]. In the case of inorganic SCs, *E*_g_^eff^ corresponds to the energy of the bandgap of the semiconductor material because the charge recombination happens from delocalized charges on the conduction and valence bands [16]. In contrast, *E*_g_^eff^ in the conventional D/A-type OSCs corresponds to the CT state energy because localized electrons on the LUMO of the acceptor and holes on the HOMO of the donor recombine [17]. In the case of the organic *pn*-homojunction SCs in this study, *E*_g_^eff^ decreased with an increase of *V*_bi_. The *E*_g_^eff^ difference between 1% and 5% is 0.20 eV, which is almost same value with the energy offset difference between 1% and 5% at the adjacent molecules at the *pn*-interface (Figure 5). The result indicates that the charge recombination in the organic *pn*-homojunction SCs happens from localized holes and electrons at the two adjacent molecules. The mechanism is similar to that of the CT state recombination in the conventional D/A-type OSCs, even though the devices in this study were fabricated using a single host material and a doping technique resembling the inorganic *pn*-homojunction SCs.

## 3. Conclusions

In summary, the temperature dependence of *J–V* characteristics and energy structure measurement revealed that the increase in *J*_SC_ and decrease in *V*_OC_ with an increase in doping concentration in the organic *pn*-homojunction SC devices was the result of the acceleration of charge separation and the change in energy of the recombination center. The charge separation mechanism in the device is that the localized exciton and CT state are separated by the energy offset between adjacent molecules, and the recombination happens from the localized charge carrier at two adjacent molecules. These mechanisms are similar to those of conventional D/A-type OSCs, not to those of the inorganic SCs. In the case of inorganic *pn*-homojunction, larger *V*_bi_ formed by higher doping concentration leads to both higher *J*_SC_ and *V*_OC_ [1]. The same tendency is favorable to obtain high efficiency in organic *pn*-homojunction SCs. The primordial difference in the photoconversion process between inorganic and organic *pn*-homojunction SCs comes from the delocalized and localized nature of charges in inorganic and organic semiconductors, respectively. Recently, some organic semiconductor materials showed a band-like nature of charges [18,19]. Utilizing these kinds of materials as hosts in organic *pn*-homojunction SCs could lead to the realization of direct free charge formation and band-to-band recombination.

## Supplementary Materials

The following are available online at https://www.mdpi.com/1996-1944/13/7/1727/s1, Figure S1: chemical structure, schematic of the device, energy levels; Figure S2: *J–V* curves; Figure S3: EQE and absorption spectra; Figure S4: WF as a function of the thickness; Figure S5: PYS spectra; Figure S6: light intensity dependence of *J*_SC_ Table S1: summary of the device performances.

## References

[1] Battaglia C., Cuevas A. (2016). High-efficiency crystalline silicon solar cells: Status and perspectives. Energy Environ. Sci..

[2] Clarke T.M., Durrant J.R. (2010). Charge photogeneration in organic solar cells. Chem. Rev..

[3] Hiramoto M., Kubo M., Shinmura Y., Ishiyama N., Kaji T., Sakai K., Ohno T., Izaki M. (2014). bandgap science for organic solar cells. Electronics.

[4] Izawa S., Perrot A., Lee J.H., Hiramoto M. (2019). Organic *pn* homojunction solar cell. Org. Electron..

[5] Izawa S., Nakano K., Suzuki K., Hashimoto K., Tajima K. (2015). Dominant effects of first monolayer energetics at donor/acceptor interfaces on organic photovoltaics. Adv. Mater..

[6] Shintaku N., Hiramoto M., Izawa S. (2018). Effect of trap-assisted recombination on open-circuit voltage loss in phthalocyanine/fullerene solar cells. Org. Electron..

[7] Riedel I., Dyakonov V. (2004). Influence of electronic transport properties of polymer-fullerene blends on the performance of bulk heterojunction photovoltaic devices. Phys. Status Solidi Appl. Res..

[8] Gao F., Tress W., Wang J., Inganäs O. (2015). Temperature dependence of charge carrier generation in organic photovoltaics. Phys. Rev. Lett..

[9] Yamamoto S., Orimo A., Ohkita H., Benten H., Ito S. (2012). Molecular understanding of the open-circuit voltage of polymer:Fullerene solar cells. Adv. Energy Mater..

[10] Izawa S., Shintaku N., Hiramoto M. (2018). Effect of band bending and energy level alignment at the donor/acceptor interface on open-circuit voltage in organic solar cells. J. Phys. Chem. Lett..

[11] Ishii H., Hayashi N., Ito E., Washizu Y., Sugi K., Kimura Y., Niwano M., Ouchi Y., Seki K. (2004). Kelvin probe study of band bending at organic semiconductor/metal interfaces: Examination of fermi level alignment. Phys. Status Solidi Appl. Res..

[12] Shintaku N., Hiramoto M., Izawa S. (2018). Doping for controlling open-circuit voltage in organic solar cells. J. Phys. Chem. C.

[13] Yamashina Y., Shinmura Y., Ishiyama N., Kaji T., Hiramoto M. (2015). Mapping of band-bending in organic *pn* –homojunctions. J. Appl. Phys..

[14] Koster L.J.A., Kemerink M., Wienk M.M., Maturová K., Janssen R.A.J. (2011). Quantifying bimolecular recombination losses in organic bulk heterojunction solar cells. Adv. Mater..

[15] Nakano K., Chen Y., Xiao B., Han W., Huang J., Yoshida H., Zhou E., Tajima K. (2019). Anatomy of the energetic driving force for charge generation in organic solar cells. Nat. Commun..

[16] Hegedus S.S., Shafarman W.N. (2004). Thin-film solar cells: Device measurements and analysis. Prog. Photovolt. Res. Appl..

[17] Burke T.M., Sweetnam S., Vandewal K., McGehee M.D. (2015). Beyond Langevin recombination: How equilibrium between free carriers and charge transfer states determines the open-circuit voltage of organic solar Cells. Adv. Energy Mater..

[18] Bussolotti F., Yang J., Yamaguchi T., Yonezawa K., Sato K., Matsunami M., Tanaka K., Nakayama Y., Ishii H., Ueno N. (2017). Hole-phonon coupling effect on the band dispersion of organic molecular semiconductors. Nat. Commun..

[19] Liu C., Minari T., Lu X., Kumatani A., Takimiya K., Tsukagoshi K. (2011). Solution-processable organic single crystals with bandlike transport in field-effect transistors. Adv. Mater..

