# Supplementary material for: Photoconversion Mechanism at the pn-Homojunction Interface in Single Organic Semiconductor
Source: Materials (Basel). 2020 Apr 7;13(7):1727. doi: 10.3390/ma13071727 (PMC7178707; doi:10.3390/ma13071727)
Supplement: Supplementary file 1 [file materials-13-01727-s001.pdf]

materials

Supplementary Materials

Photoconversion Mechanism at the *pn*-Homojunction Interface in Single Organic Semiconductor

Ji-Hyun Lee [1,2], Armand Perrot [1,3], Masahiro Hiramoto [1,2] and Seiichiro Izawa [1,2,*]

[1] Institute for Molecular Science; 5-1 Higashiyama, Myodaiji, Okazaki, Aichi 444-8787, Japan; leejihyun@ims.ac.jp (J.-H.L.); armand.perrot@etu.chimieparistech.psl.eu (A.P.); hiramoto@ims.ac.jp (M.H.)
[2] SOKENDAI, The Graduate University for Advanced Studies, 5-1 Higashiyama, Myodaiji, Okazaki, Aichi 444-8787, Japan.
[3] École Nationale Supérieure de Chimie de Paris, 11 Rue Pierre et Marie Curie, 75005 Paris, France.
* Correspondence: izawa@ims.ac.jp

Methods

The organic SC devices were fabricated on ITO-coated glass substrates (ITO thickness: 150 nm; sheet resistance: 10.3 Ω sq^{-1}; Techno Print, Fujimino, Japan). MoO_3 (Alfa Aesar, Haverhill, MA, USA), DIP (Lumtec, New Taipei City, Taiwan), Cs_2CO_3 (Sigma-Aldrich, St. Louis, MO, USA), BCP (Tokyo Chemical Industry, Tokyo, Japan) were used without further purification.

The MoO_3 hole-transporting layer (10 nm, 0.1 nm s^{-1}), doped DIP layer (100 nm, 0.1 nm s^{-1}), BCP electron-transport layer (15 nm, 0.1 nm s^{-1}), and Al electrodes (60 nm, 0.4 nm s^{-1}) were deposited via thermal evaporation under high vacuum (~10^{-5} Pa) in a vacuum evaporation system (VTS-350M, ULVAC, Kanagawa, Japan) housed in a glove box (UNICO, Suite E Dayton, NJ, USA). The MoO_3 and Cs_2CO_3 as the dopants were introduced by co-deposition with DIP.

The devices were characterized in a vacuum container for optical measurements (Epitech, Kyoto, Japan) without exposure to air. The *J*–*V* characteristics of the devices were measured under simulated solar illumination (AM 1.5, 100 mW cm^{-2}) from a solar simulator based on a 300-W Xe lamp (HAL-320, Asahi Spectra, Torrance, CA, United States) using a source meter (R6243, Advantest, Tokyo, Japan). The light intensity was calibrated with a standard silicon solar cell (CS-20, Asahi Spectra, Torrance, CA, United States) and controlled by neutral density filter (SIGMAKOKI, Tokyo, Japan). The active area of the devices was defined using a 0.04 cm^2 photomask. The EQE values of the devices were measured using a monochromator (SPG-100 ST, Shimadzu, Kyoto, Japan), a 500W Xe lamp (UI-502Q, Ushio Lighting, Tokyo, Japan), and a silicon photodiode (Hamamatsu Photonics, Hamamatsu, Japan). The *J*–*V* characteristics of the devices at various temperatures were measured in a cryostat (Janis, Woburn, MA, USA) without exposure to air.

The UV-vis absorption spectra were measured on a spectrometer (V-570, JASCO, Tokyo, Japan). The PYS spectra were measured using an (AC-2, Riken-Keiki, Tokyo, Japan). The WF was determined using a Kelvin probe (FAC-1, Riken-Keiki, Tokyo, Japan) without exposure to air. The Kelvin probe was housed in a glove box with N_2 gas (H_2O < 1 ppm, O_2 < 1 ppm).

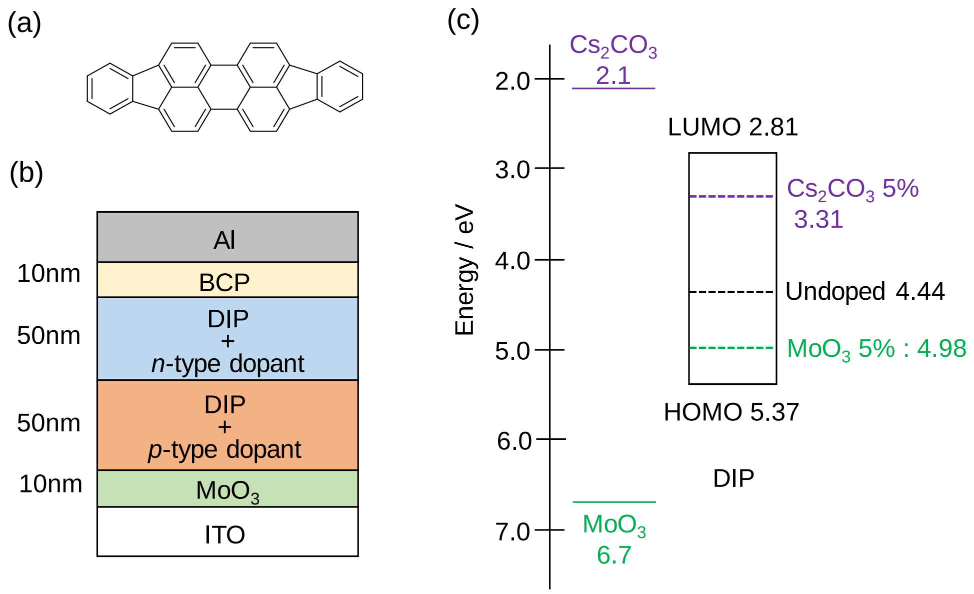

Figure S1. (**a**) Chemical structure of DIP. (**b**) Schematic of the *pn*-homojunction device. (**c**) Energy levels of DIP, MoO_3 and Cs_2CO_3. The dashed lines indicate the position of E_F of the 50 nm undoped (black), 5% MoO_3 doped (green) and 5% Cs_2CO_3 doped (purple) films on ITO substrates. The energy level of DIP is sited from Appl. Phys. Lett. 101, 233301 (2012), and HOMO and LUMO are measured by ultraviolet and inverse photoelectron spectroscopy in the paper. Reprinted with permission from S. Izawa et al., *Org. Electron.* **2019**, *71*, 45–49. Copyright 2019, Elsevier.

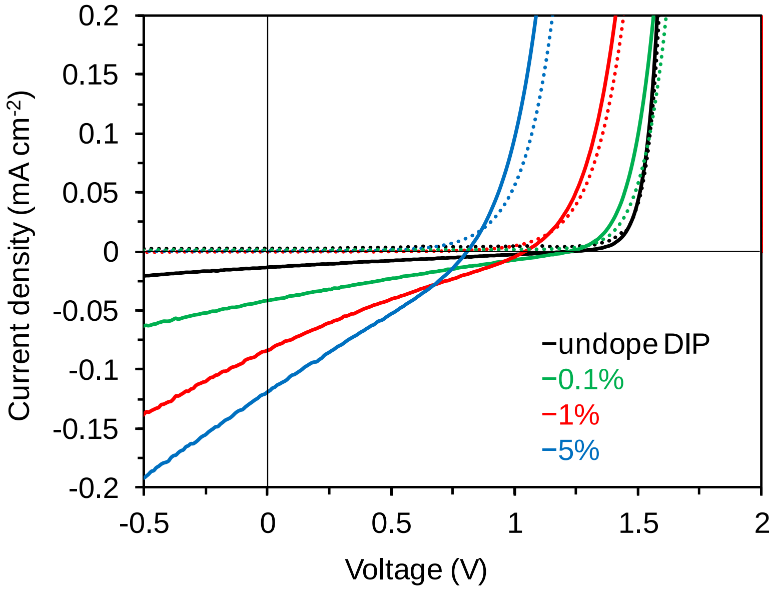

Figure S2. *J*–*V* curves of homojunction devices with 100 nm undoped layer (black) and *pn* doped DIP layers having same MoO_3 and Cs_2CO_3 concentrations of 0.1% (green), 1% (red) and 5% (blue) using DIP as a host molecule under AM 1.5 irradiation (100 mW cm^{-2}, solid lines) or in the dark (dashed lines). The same concentrations of *p*- and *n*-dopants were introduced in the active layers. Reprinted with permission from S. Izawa et al., *Org. Electron.* **2019**, *71*, 45–49. Copyright 2019, Elsevier.

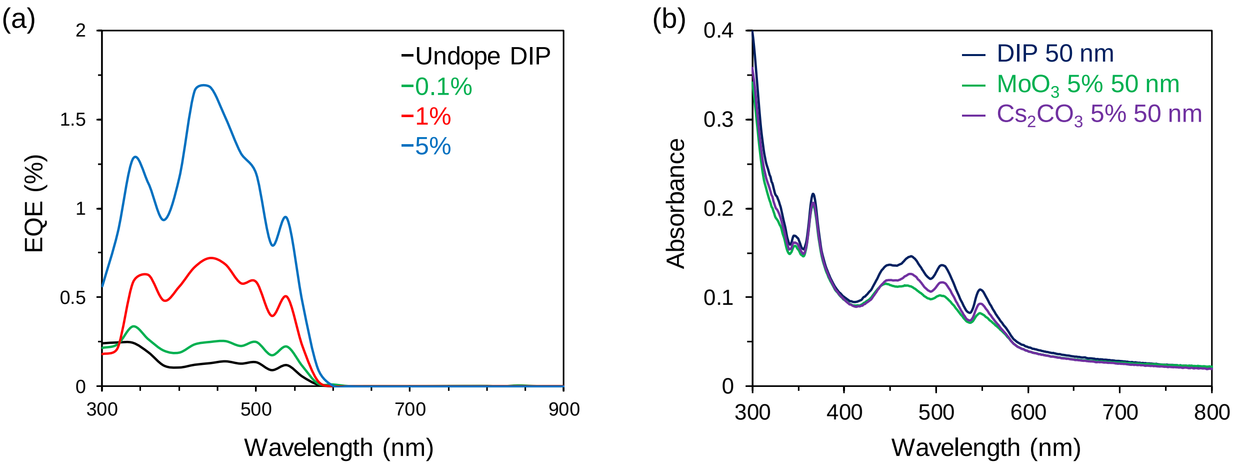

Figure S3. (**a**) EQE spectra of homojunction devices with undoped DIP layer (black), and *pn* doped DIP layers with same MoO_3 and Cs_2CO_3 concentrations of 0.1% (green), 1% (red) and 5% (blue). (**b**) Absorption spectra of undoped (dark blue), 5% MoO_3 doped (green) and 5% Cs_2CO_3 doped (purple) DIP films on quartz. The amount of DIP on quartz is equivalent to 50 nm in all the films. Reprinted with permission from S. Izawa et al., *Org. Electron.* **2019**, *71*, 45–49. Copyright 2019, Elsevier.

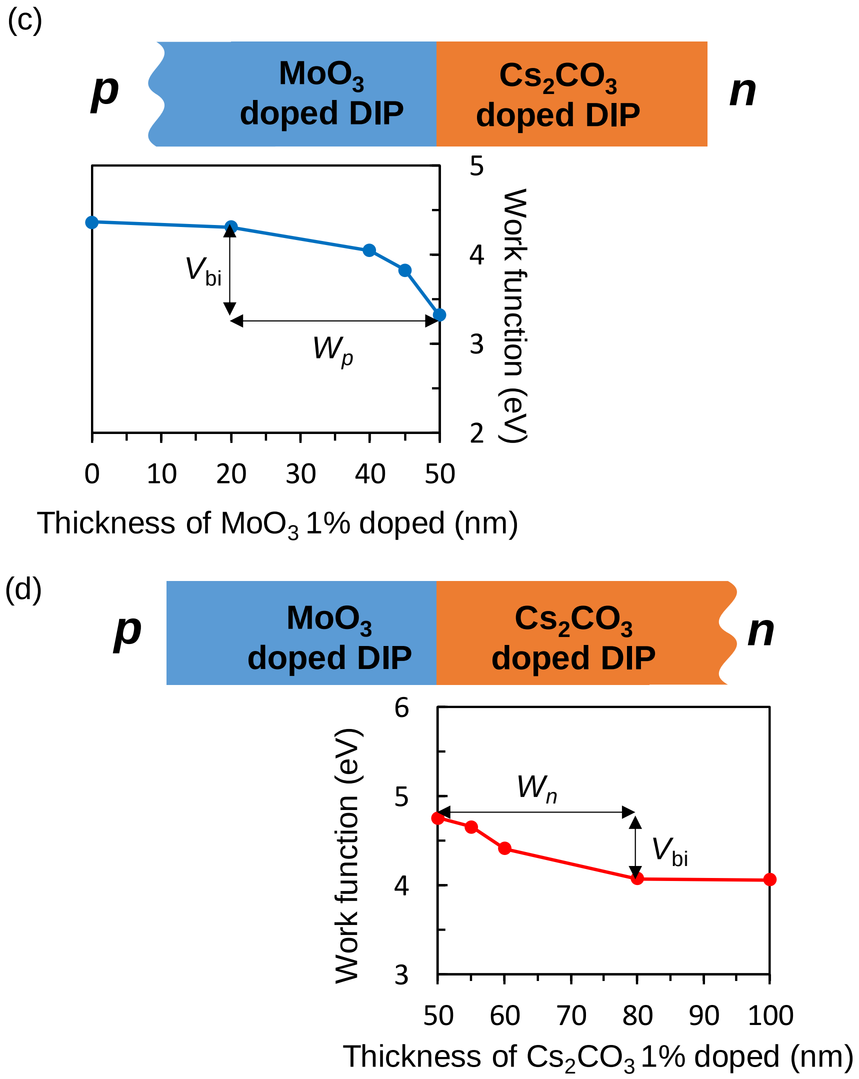

Figure S4. WF of (**a**) 1% MoO_3 doped and (**b**) 1% Cs_2CO_3 doped DIP films on the Cs_2CO_3 doped film and the MoO_3 doped film, respectively, as a function of the thickness.

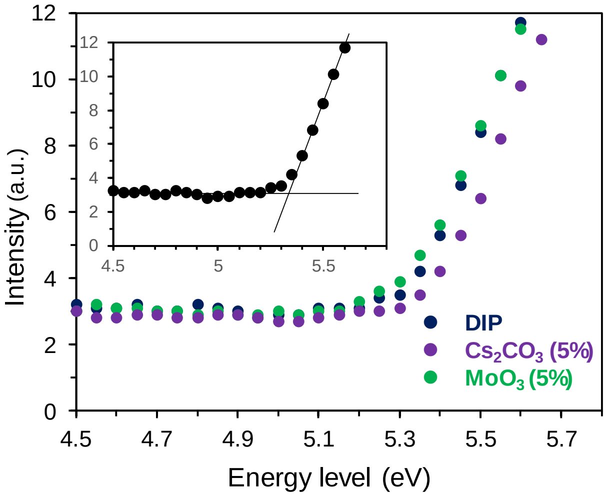

Figure S5. Photoelectron yield spectra of undoped DIP (dark blue), Cs_2CO_3 5% doped DIP (purple) and MoO_3 5% doped DIP (green) thin film. Ionic potential is estimated by approximate line of plot.

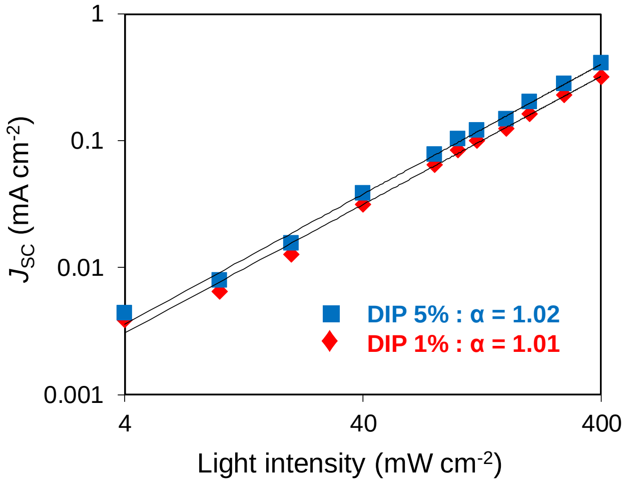

Figure S6. Light intensity dependence of J_{SC} of the devices with the 1% (red) and 5% (blue) doped DIP. The value of α shows the slope of approximate line.

Table S1. Summary of the performances of DIP devices with different doping concentrations of MoO_3 and Cs_2CO_3. Averaged values are reported. Values inside parentheses are standard deviations. Reprinted with permission from S. Izawa et al., *Org. Electron.* **2019**, *71*, 45–49. Copyright 2019, Elsevier.

Concentration (%)	Undoped	0.1	1	5
JSC (mA cm^{-2})	0.014 (0.001)	0.056 (0.020)	0.067 (0.017)	0.121 (0.008)
VOC (V)	1.13 (0.14)	1.18 (0.04)	0.98 (0.02)	0.83 (0.10)
FF (%)	25.0 (1.4)	24.4 (0.7)	24.4 (1.6)	29.5 (2.4)
PCE (%)	0.0039 (0.0006)	0.016 (0.006)	0.016 (0.005)	0.026 (0.002)

© 2020 by the authors. Licensee MDPI, Basel, Switzerland. This article is an open access article distributed under the terms and conditions of the Creative Commons Attribution (CC BY) license (http://creativecommons.org/licenses/by/4.0/).
